# Association between alcohol consumption and hepatic fibrosis in Chinese adult males with metabolic dysfunction-associated steatotic liver disease

**DOI:** 10.3389/fmed.2025.1572853

**Published:** 2025-04-28

**Authors:** Mao Li, Bin Yu, Xiaoli Zhang, Jia Pan, Lei Tang, Yi Zhang, Ruixin Wang, Honglian Zeng, Shujuan Yang

**Affiliations:** ^1^Department of Health Management Centre, Clinical Medical College and Affiliated Hospital of Chengdu University, Chengdu University, Chengdu, Sichuan, China; ^2^West China School of Public Health and West China Fourth Hospital, Sichuan University, Chengdu, Sichuan, China; ^3^North Sichuan Medical College, Nanchong, Sichuan, China

**Keywords:** metabolic dysfunction-associated steatotic liver disease, hepatic fibrosis, fibrosis-4 index, alcohol consumption, metabolic and alcohol-related liver disease

## Abstract

**Background:**

The impact of moderate drinking on the risk of liver fibrosis in non-alcoholic fatty liver disease (NAFLD) remains controversial worldwide. Notably, China, with the fastest-growing incidence of NAFLD and the highest number of alcohol-attributable deaths globally, has relatively few studies addressing this issue. This study aimed to explore the association between alcohol consumption and liver fibrosis in Chinese men with metabolic dysfunction-associated steatotic liver disease (MASLD).

**Methods:**

We recruited 4,683 male employees diagnosed with MASLD from southwest China, including 4,287 with pure MASLD and 396 with metabolic and alcohol-related liver disease (MetALD) who consumed increased alcohol (30-60 g/d). Advanced fibrosis was defined as a fibrosis-4 index (FIB-4) ≥ 2.67, and FIB-4 ≥ 1.30 indicated an intermediate/high probability of hepatic fibrosis. Logistic regression models were used to assess the association between alcohol consumption and hepatic fibrosis, and analyze the modification effect of body mass index (BMI) and waist-to-hip ratio (WHR) on the association. Propensity score matching method was used to test the robustness of the regression results.

**Results:**

Compared with non-drinkers, both moderate (OR = 3.02, 95% CI: 1.16-10.31) and increased alcohol consumption (OR = 4.64, 95% CI: 1.60-16.82) were significantly associated with an increased risk of advanced fibrosis in males with MASLD. Additionally, moderate (OR = 1.33, 95% CI: 1.07-1.66) and increased drinking (OR = 1.74, 95% CI: 1.28-2.34) were associated with intermediate/high probability of hepatic fibrosis, with similar results from logistic regression analysis in propensity score-matched cases. Trend analysis revealed the risk of hepatic fibrosis increased with increasing alcohol intake (FIB-4 ≥ 1.30, *p* for trend < 0.001; FIB-4 ≥ 2.67, *p* for trend = 0.007). Further subgroup analysis showed that the association between moderate drinking and intermediate/high probability of hepatic fibrosis was predominantly observed in males with BMI ≥ 23 kg/m^2^ (OR = 1.35, 95% CI: 1.08-1.69) and those with WHR ≥ 0.9 (OR = 1.40, 95% CI: 1.11-1.78).

**Conclusion:**

In China, moderate alcohol intake may heighten the risk of hepatic fibrosis in males with MASLD who are overweight/obese or have abdominal obesity. Moreover, males with MetALD may have a higher risk of fibrosis compared to those with pure MASLD.

## 1 Introduction

Non-alcoholic fatty liver disease (NAFLD) is a major contributor to chronic liver disease, affecting over 25% of the global population (1). Geographically, China has the highest incidence rate of NAFLD globally, at 59.4 per 1,000 person-years (2). Over the past 20 years, the pooled prevalence of NAFLD among Chinese adults was 29.6%, with a higher rate in males (34.8%) than in females (23.5%) (3). NAFLD can progress to non-alcoholic steatohepatitis, fibrosis, cirrhosis, and hepatocellular carcinoma, and it also increases the risk of atherosclerotic cardiovascular disease and chronic kidney disease (4, 5). Therefore, NAFLD in China, particularly among males, is becoming increasingly severe. Notably, hepatic fibrosis is widely recognized as a key predictor of poor prognosis in individuals with NAFLD (6).

In 2023, a multi-society Delphi consensus statement on a new fatty liver disease nomenclature was published, introducing the term metabolic dysfunction-associated steatotic liver disease (MASLD) and effectively retiring the term NAFLD (7). Additionally, beyond pure MASLD, a new classification termed metabolic and alcohol-related liver disease (MetALD) was introduced to describe individuals with MASLD who have increased alcohol intake, averaging 20-50 g/day for females and 30-60 g/day for males. The updated nomenclature and diagnostic criteria have garnered broad support and are non-stigmatizing. By highlighting the central role of metabolic dysfunction in disease pathogenesis, MASLD offers a clearer framework for understanding, treating, and preventing the condition, thereby enhancing disease awareness and facilitating more accurate patient identification.

Alcohol consumption is highly prevalent worldwide, with the prevalence of current drinking among adults reaching 47% in 2017 (8). However, there is no consensus regarding the impact of light or moderate alcohol consumption on liver fibrosis in NAFLD or MASLD (9–12). A meta-analysis comprising eight cross-sectional surveys from the United States, South Korea, Sweden, Japan, Australia, and Malaysia revealed that NAFLD patients who engaged in modest alcohol consumption exhibited a significantly lower risk of advanced liver fibrosis compared to those who abstained from alcohol (10). In contrast, a cohort study from South Korea found that moderate alcohol consumption was associated with an increased risk of liver fibrosis in NAFLD patients, which was defined by non-invasive liver fibrosis markers (11). Additionally, a small-sample cohort study from Sweden showed that NAFLD patients consuming moderate amounts of alcohol were at increased risk for significant fibrosis progression and development of cirrhosis-related complications (12). These conflicting results may be related to genetic and ethnic differences, body composition, as well as the types of alcohol consumed in different regions (11, 13, 14). China, the country with the highest number of deaths attributed to alcohol consumption worldwide (13), has witnessed a yearly increase in the prevalence of alcohol drinking, with a significantly higher rate among men (33%) than women (2%) (15). However, for the Chinese population, there are very limited epidemiological and clinical studies available.

To fill this research gap, we aim to investigate the association between alcohol consumption and the risk of hepatic fibrosis among adult males with MASLD in Southwest China. Furthermore, we will investigate the potential moderating effects of body mass index (BMI) and waist-to-hip ratio (WHR) on this association. A better understanding of the effects of alcoholic beverage intake on MASLD fibrosis could offer a scientific basis for targeted management strategies in the Chinese population.

## 2 Materials and methods

### 2.1 Study design and population

This cross-sectional study recruited 21,499 Chinese male railroad employees from Sichuan Province, Guizhou Province, and Chongqing. All participants underwent health examinations at the Affiliated Hospital of Chengdu University between January 2020 and December 2020.

The study included male employees who met the diagnostic criteria for MASLD (7). The following employees were excluded: (1) those with missing or insufficient clinical data (e.g., blood pressure, fasting glucose, blood lipid levels, or body mass index); (2) those with hepatitis B or C virus infection; (3) those with liver cancer or a history of liver surgery; (4) those with a history of severe diseases (e.g., renal or liver failure, malignancy); (5) those with an average daily alcohol intake exceeding 60 g. Finally, 4,287 male employees with pure MASLD and 396 with MetALD (increased alcohol intake, 30-60 g/day) were included in this analysis.

### 2.2 Data collection

The health examination consisted of a comprehensive assessment, including a face-to-face survey, physical examination, and laboratory tests. Face-to-face surveys conducted by uniformly trained physicians collected data from employees on basic demographic information (including age, gender, and marital status), health behaviors (including smoking and alcohol intake), chronic disease history (such as hypertension and diabetes), and medication use. Participants’ height, weight, waist circumference (WC), and hip circumference were measured using standard equipment by trained medical staff. The BMI was calculated as weight divided by height squared (kg/m^2^). The WHR was also calculated. Abdominal obesity was defined as WHR ≥ 0.9 or WC ≥ 90 cm in men (16, 17). Blood pressure was measured by well-trained nurses using an electronic sphygmomanometer after participants had rested for at least 5 min in a seated position. Blood tests were performed by laboratory technicians at the Clinical Laboratory of the Affiliated Hospital of Chengdu University. All participants fasted for at least 8 h before blood collection. Venous blood was collected to measure fasting plasma glucose (FPG), plasma triglycerides (TG), low-density lipoprotein cholesterol (LDL-C), high-density lipoprotein cholesterol (HDL-C), high-sensitivity C-reactive protein (hs-CRP), serum uric acid (SUA), aspartate aminotransferase (AST), alanine transaminase (ALT), and other biochemical indicators.

All employees underwent abdominal ultrasonography performed by experienced and uniformly trained imaging physicians using digital ultrasonic diagnostic systems (Clear Vue 580, Philips Ultrasound Inc., United States). Hepatic steatosis was assessed in accordance with the guidelines of the Chinese Association for the Study of Liver Disease (CASLD) (18).

### 2.3 Definition of MASLD and MetALD

The diagnosis of MASLD was based on criteria endorsed by an international expert panel (7, 19). It includes evidence of hepatic steatosis, along with at least one of the following cardiometabolic risk factors: (1) BMI ≥ 23 kg/m^2^ (Asians), or waist circumference > 90 cm in men; (2) FPG ≥ 5.6 mmol/L or type 2 diabetes, or treatment for type 2 diabetes; (3) blood pressure ≥ 130/85 mmHg or treatment with antihypertensive medication; (4) TG ≥ 1.70 mmol/L or treatment with lipid-lowering treatment; (5) plasma HDL-C < 1.0 mmol/L for men, or lipid lowering treatment. In this Asian population, overweight/obesity was defined as a BMI ≥ 23 kg/m^2^, also referred to as non-lean. Lean individuals were defined as having a BMI < 23 kg/m^2^. The WHO criteria were used to define diabetes, encompassing a self-reported history of diabetes, use of anti-diabetic medication, or FPG ≥ 7.0 mmol/L (20). Although other cardiometabolic risk factors, such as 2-h post-load glucose levels ≥ 7.8 mmol/L and glycated hemoglobin (HbA1c) ≥ 5.7% were also considered, these data were not available in our dataset. Individuals with MASLD and an alcohol intake of 30-60 g/day were categorized as having MetALD, a distinct clinical category, while those with an intake of 0-29 g/day were classified as having pure MASLD.

### 2.4 Exposure

Alcohol consumption was categorized into three groups: no consumption, moderate consumption (1-29 g/day), and increased consumption (30-60 g/day) (19, 21). Former drinkers were classified as moderate or increased drinkers based on their past alcohol consumption, considering that individuals often cease drinking upon experiencing severe hepatic steatosis.

### 2.5 Outcome

The fibrosis-4 index (FIB-4) was used to evaluate the probability of fibrosis in MASLD (22) or MetALD (23). As previously published, the FIB-4 index was calculated using age, serum levels of AST, ALT, and platelet (PLT) count (24). A FIB-4 index of ≥ 2.67 was used to define advanced fibrosis, while a FIB-4 score of ≥ 1.30 indicated an intermediate/high probability of hepatic fibrosis (25, 26). Additionally, we calculated the value of the NAFLD Fibrosis Score (NFS) (24).

### 2.6 Covariates

The selection of covariates was informed by previously published researches (27–30). In this study, the adjusted covariates included age, diabetes mellitus, hypertension, hyperuricemia, TG, HDL-C, LDL-C, and BMI. Hypertension was defined as a systolic blood pressure (SBP) ≥ 140 mmHg or a diastolic blood pressure (DBP) ≥ 90 mmHg, or current use of antihypertensive medication (31). Hyperuricemia was defined as SUA > 7.0 mg/dl in men (32).

### 2.7 Statistical analysis

The characteristics of the study sample were described according to alcohol consumption categories. Differences in demographic characteristics, lifestyle information, laboratory indicators were analyzed by one-way ANOVA (when the data met normal distribution) and Kruskal-Wallis test (when the data did not meet normal distribution) for continuous variables, and by Chi-square test and Fisher’s Exact test for categorical variables. Multiple logistic regression was used to explore the association between alcohol consumption and the risk of hepatic fibrosis. Specifically, we evaluated the risk of hepatic fibrosis at two FIB-4 thresholds: FIB-4 ≥ 2.67 and FIB-4 ≥ 1.30. Covariates were adjusted in a stepwise manner based on previously published studies (27–30). We employed a stepwise regression analysis strategy, which included no covariate adjustments (Model 1), adjustment for age (Model 2), adjustment for age and common chronic disease indicators (Model 3), and adjustment for age, common chronic disease indicators, and BMI (Model 4). The P for trend was calculated by modeling alcohol consumption categories as a linear term in the regression models. Subsequently, subgroup analyses were conducted based on BMI and WHR to investigate their modifying effects.

Finally, we conducted a sensitive analysis using pairwise propensity score matching (PSM) to address potential confounding. Specifically, PSM was performed between two pairs: (1) non-drinking and moderate drinking groups, and (2) non-drinking and increased drinking groups. Propensity scores were calculated based on covariates including age, common chronic disease indicators and BMI. One-to-one nearest neighbor matching was conducted with a caliper width of 0.1-0.2, and balance was assessed using standardized mean differences (SMD) of less than 0.1 for all covariates. Logistic regression analysis was then performed on the matched datasets to assess the association between alcohol consumption and hepatic fibrosis. All statistical analyses were performed using R Studio (version 4.2.1) with a two-sided significance level of 0.05.

## 3 Results

### 3.1 Baseline characteristics

Among the 21,499 Chinese male employees recruited in this study, 5,048 were identified as having fatty liver through ultrasound detection. Of these, 5,007 had at least one cardiometabolic risk factor. After excluding 324 individuals with alcohol intake exceeding 60 g/day, the final analysis included 4,287 males with pure MASLD (1,307 non-drinkers and 2,980 moderate drinkers) and 396 males with MetALD. Table 1 shows significant differences in baseline characteristics among MAFLD males across different alcohol intake groups, including age, BMI, WHR, TG, TC, LDL-C, HDL-C, FBG, SUA, PLT, ALT, albumin, marital status, smoking habits, and prevalence of diabetes and hypertension (all *p* < 0.01). With increasing alcohol consumption, the non-invasive fibrosis markers FIB-4 and NFS exhibited an upward trend.

**TABLE 1:** Basic characteristics of male employees with MASLD categorized by alcohol intake.

Characteristics	Total (*n* = 4,683)	Non-drinkers (*n* = 1,307)	Moderate drinkers (*n* = 2,980)	Increased drinkers (*n* = 396)	*P*-value
**Sociodemographics**					
Age (years)	44.00 (31.00, 50.00)	44.00 (30.00, 50.00)	43 (31.00, 49.00)	49.00 (45.00, 53.00)	<0.001
Married (%)	3,997 (85.35)	1,075 (82.25)	2,542 (85.3)	380 (95.96)	<0.001
**Lifestyle behaviors**					
Current smokers (%)	2,974 (63.51)	697 (53.33)	1,982 (66.51)	295 (74.49)	<0.001
**Clinical variables**					
Diabetes (%)	844 (18.02)	231 (17.67)	506 (16.98)	107 (27.02)	<0.001
Hypertension (%)	1,997 (42.64)	478 (36.57)	1,294 (43.42)	225 (56.82)	<0.001
BMI (kg/m^2^)	27.46 (25.66, 29.55)	27.45 (25.64, 29.62)	27.53 (25.76, 29.55)	27.19 (25.17, 28.95)	<0.001
WC (cm)	94.00 (89.00, 99.00)	93.00 (89.00, 99.00)	94.00 (89.00, 100.00)	94.00(90.00, 99.00)	0.319
WHR	0.94 (0.91, 0.97)	0.93 (0.90, 0.97)	0.94 (0.91, 0.97)	0.95 (0.92, 0.98)	<0.001
TG (mmol/L)	2.34 (1.68, 3.46)	2.16 (1.57, 3.08)	2.43 (1.70, 3.55)	2.47 (1.81, 4.08)	<0.001
TC (mmol/L)	5.11 (4.51, 5.77)	4.96 (4.42, 5.58)	5.17 (4.54, 5.80)	5.34 (4.71, 5.96)	<0.001
LDL-C (mmol/L)	3.17 (2.74, 3.63)	3.09 (2.71, 3.51)	3.21 (2.76, 3.67)	3.23(2.75, 3.68)	<0.001
HDL-C (mmol/L)	1.10 (0.95, 1.27)	1.07 (0.93, 1.23)	1.10 (0.95, 1.28)	1.17 (1.00, 1.36)	<0.001
FBG (mmol/L)	5.59 (5.20, 6.31)	5.53 (5.16, 6.11)	5.59 (5.19, 6.27)	5.97 (5.39, 7.19)	<0.001
hs-CRP (mg/L)	2.16 (1.34, 3.68)	2.19 (1.36, 3.79)	2.13 (1.33, 3.65)	2.23 (1.30, 3.61)	0.482
SUA (mmol/L)	432.00 (375.00, 497.00)	431.00 (370.00, 496.00)	433.00 (378.00, 499.00)	424.00 (363.00, 481.00)	<0.003
PLT (×10^9^/L)	222.00 (184.00, 262.00)	224.00 (185.00, 265.00)	224.00 (186.00, 262.00)	214.00 (174.00, 251.75)	<0.001
ALT (U/L)	42.00 (29.00, 62.00)	43.00 (30.00, 65.00)	43.00 (30.00, 63.00)	37.00 (26.00, 54.00)	<0.001
AST (U/L)	27.00 (22.00, 35.00)	26.00 (21.00, 34.00)	27.00 (22.00, 35.00)	27.00 (21.25, 36.00)	0.072
Albumin (g/L)	46.10 (44.50, 47.70)	46.00 (44.50, 47.60)	46.20 (44.60, 47.70)	45.60 (44.30, 47.20)	<0.001
**Outcome variables**					
FIB-4	0.80 (0.55, 1.10)	0.76 (0.53, 1.02)	0.78 (0.54, 1.09)	1.06 (0.77, 1.43)	<0.001
FIB-4 ≥ 1.30 (%)	746 (15.93)	164 (12.55)	457 (15.34)	125 (31.57)	<0.001
FIB-4 ≥ 2.67 (%)	53 (1.13)	4 (0.31)	33 (1.11)	16 (4.04)	<0.001
NFS	–2.21 (–3.20, –1.28)	–2.40 (–3.27, –1.45)	–2.24 (–3.24, –1.31)	–1.46 (–2.33, –0.79)	<0.001
NFS ≥ –1.455 (%)	1,385 (29.58)	328 (25.10)	860 (28.86)	197 (49.75)	<0.001
NFS ≥ 0.676 (%)	32 (0.68)	2 (0.15)	23 (0.77)	7 (1.77)	0.002

Data are presented as Median (P25, P75) or n (%). MASLD, metabolic dysfunction-associated steatotic liver disease; BMI, body mass index; WC, waist circumference; WHR, waist-to-hip ratio; TG, triglycerides; TC, total cholesterol; LDL-C, low-density lipoprotein cholesterol; HDL-C, high-density lipoprotein cholesterol; FBG, fasting plasma glucose; hs-CRP, hypersensitive C-reactive protein; SUA, serum uric acid; PLT, platelet; ALT, alanine transaminase; AST, aspartate aminotransferase; FIB-4, Fibrosis-4 Index; NFS, NAFLD fibrosis score.

### 3.2 The association between alcohol consumption and hepatic fibrosis in MASLD

Among male employees with MASLD, the prevalence of advanced fibrosis was highest among those with increased alcohol consumption (4.04%), followed by moderate drinkers (1.11%), while non-drinkers had the lowest rate (0.31%, *p* < 0.001) (Table 1). After adjusting for potential confounding factors, the multivariable logistic regression model indicated that, compared to non-drinkers, both moderate (OR = 3.02, 95% CI: 1.16-10.31) and increased alcohol consumption (OR = 4.64, 95% CI: 1.60-16.82) were associated with a higher risk of advanced fibrosis in males with MASLD (Table 2). Additionally, the risk of advanced fibrosis increased significantly with increasing daily alcohol intake (*p* for trend = 0.007). Subsequently, we designated FIB-4 ≥ 1.30 as the outcome variable and found that both moderate (OR = 1.33, 95% CI: 1.07-1.66) and increased alcohol consumption (OR = 1.74, 95% CI: 1.28-2.34) were significantly associated with an intermediate/high probability of hepatic fibrosis (Table 2). Tests for trend were also significant (*p* for trend < 0.001).

**TABLE 2:** The association between alcohol consumption and hepatic fibrosis score (FIB-4) in males with MASLD.

Alcohol consumption	Model 1		Model 2		Model 3		Model 4	
	**OR (95%CI)**	***P*-value**	**OR (95%CI)**	***P*-value**	**OR (95%CI)**	***P*-value**	**OR (95%CI)**	***P*-value**
**FIB-4 ≥ 1.30**								
No drinking	1.00 (ref)		1.00 (ref)		1.00 (ref)		1.00 (ref)	
Moderate drinking	1.26 (1.04,1.53)	0.017	1.41 (1.15,1.75)	0.001	1.34 (1.08,1.66)	0.008	1.33 (1.07,1.66)	0.009
Increased drinking	3.21(2.46,4.20)	<0.001	2.02 (1.51,2.70)	<0.001	1.73 (1.28,2.34)	<0.001	1.74 (1.28,2.34)	<0.001
P for trend^a^		<0.001		<0.001		<0.001		<0.001
**FIB-4 ≥ 2.67**								
No drinking	1 (ref)		1 (ref)		1 (ref)		1 (ref)	
Moderate drinking	3.65 (1.45,12.25)	0.015	4.05 (1.60,13.66)	0.009	2.98 (1.15,10.15)	0.044	3.02 (1.16,10.31)	0.041
Increased drinking	13.72 (5.00,48.10)	<0.001	8.62 (3.12,30.39)	<0.001	4.86 (1.69,17.55)	0.007	4.64 (1.60,16.82)	0.009
P for trend^a^		<0.001		<0.001		0.004		0.007

^a^P for trend was calculated by modeling alcohol consumption categories as a linear term in the regression models. Model 1 No adjusted. Model 2 Adjusted for age. Model 3 Adjusted for age, diabetes mellitus, hypertension, TG, HDL-C, LDL-C and hyperuricemia. Model 4 Adjusted for age, diabetes mellitus, hypertension, TG, HDL-C, LDL-C, hyperuricemia and BMI. FIB-4, fibrosis-4 index; OR, odds ratios; CI, confidence intervals; ref, reference.

### 3.3 Subgroup analysis

**FIGURE 1:**
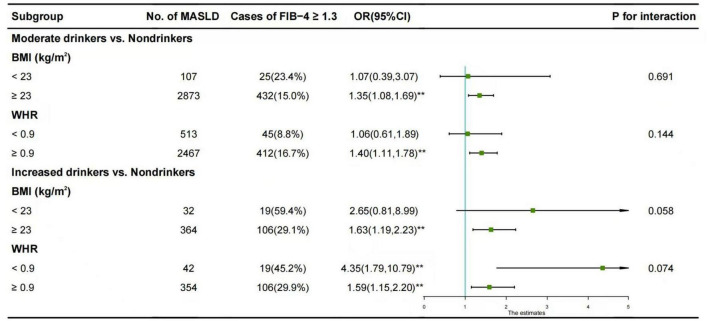
Subgroup analysis of the association between alcohol consumption and FIB-4 ≥ 1.30 in males with MASLD. The reference group was non-drinkers. Horizontal lines represent 95% confidence intervals. Adjustment for age, diabetes mellitus, hypertension, hyperuricemia, TG, LDL-C, and HDL-C. *p < 0.05, **p < 0.01. OR, odds ratios; CI, confidence intervals; BMI, body mass index; WHR, waist-to-hip ratio; vs., versus.

The results of the subgroup analysis are depicted in Figure 1. The association between moderate alcohol consumption and FIB-4 ≥ 1.30 was predominantly observed in males with BMI ≥ 23 kg/m^2^ (OR = 1.35, 95% CI: 1.08-1.69) and in those with WHR ≥ 0.9 (OR = 1.40, 95% CI: 1.11-1.78).

### 3.4 Sensitive analysis

PSM between non-drinkers and moderate drinkers resulted in 1,305 matched pairs. Balance was achieved for all covariates (SMD < 0.1) except for TG. Statistical tests confirmed no significant differences between the groups for these covariates (*p* > 0.05) (Table 3), except for TG. Multivariate-adjusted regression analysis indicated that moderate drinking was associated with an intermediate/high probability of hepatic fibrosis compared to non-drinking (OR = 1.47, 95% CI: 1.15-1.89) (Table 4A).

**TABLE 3:** Clinical characteristics after propensity score matching.

Characteristics	Non-drinkers vs. moderate drinkers			Non-drinkers vs. increased drinkers		
	**Non-drinkers** **(*n* = 1,305)**	**Moderate drinkers (*n* = 1,305)**	***P*-value**	**Non-drinkers** **(*n* = 356)**	**Increased drinkers (*n* = 356)**	***P*-value**
Age (years)	29.00 (26.00,34.25)	30.00 (27.00,37.00)	0.436	49.00 (44.00,54.00)	49.00 (44.00,52.00)	0.204
Diabetes (%)	230 (17.62)	222 (17.01)	0.679	101 (28.37)	96 (26.97)	0.675
Hypertension (%)	478 (36.63)	507 (38.85)	0.242	199 (55.90)	192 (53.93)	0.598
BMI (kg/m^2^)	28.25 (26.16,30.69)	27.96 (26.07,29.96)	0.708	27.16 (25.63,29.05)	27.32 (25.26,29.10)	0.628
TG (mmol/L)	2.08 (1.52,3.02)	2.29 (1.65,3.32)	<0.001	2.38 (1.66,3.70)	2.45 (1.84,4.03)	0.075
LDL-C (mmol/L)	3.08 (2.68,3.49)	3.12 (2.69,3.61)	0.068	3.23 (2.77,3.60)	3.22 (2.75,3.66)	0.888
HDL-C (mmol/L)	1.09 (0.93,1.23)	1.06 (0.92,1.22)	0.784	1.13 (0.96,1.31)	1.13 (0.99,1.31)	0.480
hs-CRP (mg/L)	2.27 (1.41,3.88)	2.21 (1.35,3.70)	0.135	2.12 (1.39,3.57)	2.27 (1.37,3.63)	0.411
SUA (mmol/L)	466.50 (412.00,539.00)	465.00 (412.00,526.00)	0.476	428.00 (364.00,486.00)	423.00 (362.25,480.75)	0.559

Data are presented as Median (P25, P75) or n (%). BMI, body mass index; TG, triglycerides; LDL-C, low-density lipoprotein cholesterol; HDL-C, high-density lipoprotein cholesterol; hs-CRP, hypersensitive C-reactive protein; SUA, serum uric acid.

**TABLE 4:** The association between moderate alcohol consumption and liver fibrosis score (FIB-4 ≥ 1.30) in men with MASLD after propensity score matching.

Alcohol consumption	Crude model		Adjusted model^a^	
	**OR (95%CI)**	***P*-value**	**OR (95%CI)**	***P*-value**
**(A) Moderate drinking vs. no drinking (matched sample size: 1,305 per group).**				
No drinking	1 (ref)		1 (ref)	
Moderate drinking	1.36 (1.09,1.70)	0.006	1.47 (1.15,1.89)	0.002
**(B) Increased drinking vs. no drinking (matched sample size: 356 per group)**				
No drinking	1 (ref)		1 (ref)	
Increased drinking	1.42 (1.02,2.00)	0.040	1.64 (1.13,2.38)	0.009

^a^Adjusted for age, diabetes mellitus, hypertension, TG, HDL-C, LDL-C, hyperuricemia and BMI. FIB-4, fibrosis-4 index; OR, odds ratios; CI, confidence intervals; ref, reference.

Matching between non-drinkers and increased drinkers yielded 356 matched pairs. Balance was achieved for all covariates (SMD < 0.1), with no significant differences between the groups (*p* > 0.05) (Table 3). Multivariate-adjusted regression analysis showed that increased drinking was associated with an intermediate/high probability of hepatic fibrosis compared to non-drinking (OR = 1.64, 95% CI: 1.13-2.38) (Table 4B).

## 4 Discussion

In this cross-sectional study of the Chinese population, we found that, compared to non-drinkers, even moderate alcohol intake was associated with an increased risk of hepatic fibrosis in males with MASLD, predominantly in those who are overweight/obese or have abdominal obesity. Additionally, increased drinking was associated with hepatic fibrosis in males with MASLD, and the risk of hepatic fibrosis increased with increasing alcohol intake.

Our study revealed a correlation between moderate alcohol consumption and an elevated risk of advanced fibrosis in males with MASLD. Although similar studies based on MASLD are limited, relevant research based on NAFLD exists. So far, the debate continues regarding whether moderate alcohol consumption heightens the risk of liver fibrosis in individuals with NAFLD (9–12). Firstly, this may partly be attributed to differences in the types of alcoholic beverages consumed across different regions and ethnicities. Globally, the most consumed types of alcohol are spirits (44.8%), beer (34.3%), and wine (11.7%) (13). In Southeast Asia, spirits account for 87.9% of alcohol consumption, while beer is the most consumed beverage in the Americas (53.8%) and Europe (40.0%), wine consumption is highest in Europe (29.8%). Different types of alcoholic beverages contain various flavor compounds, some of which may offer additional health impacts (33). A biopsy-based study from Australia demonstrated that modest alcohol consumption (1-70 g per week), particularly exclusive wine drinking but not beer drinking, was associated with lower fibrosis in NAFLD patients compared to lifetime abstainers (34). This association may be attributed to the antioxidants in wine, such as polyphenols, which possess anti-inflammatory and antioxidant properties, improve insulin resistance, and enhance lipid profiles (35). However, in China, wine consumption accounts for only 3%, while spirits, predominantly Chinese baijiu, account for 67%, followed by beer at 30% (33). While some animal and *in vitro* studies have identified over 1,874 flavor compounds in Chinese baijiu, such as pyrazines, esters, and terpenes, which exhibit antioxidant, anti-inflammatory, and lipid metabolism modulation effects, their low concentrations may still be insufficient to counteract the harmful effects of alcohol on NAFLD or MASLD (33). Secondly, the impact of moderate alcohol consumption on the prognosis of NAFLD may also be related to variations in alcohol metabolism genes among different ethnic populations. Acetaldehyde, a toxic metabolite of ethanol, is metabolized to non-toxic acetate by aldehyde dehydrogenase (ALDH). Studies have found that 36-45% of East Asians (particularly in China, Japan, and South Korea) carry an inactivating mutation in the ALDH2 gene, which leads to impaired acetaldehyde metabolism (36). Whereas, this mutation is less frequent in other regions, such as Europe and the Americas. This may partly explain the association between moderate alcohol consumption and liver fibrosis in NAFLD or MASLD observed in our study as well as in studies from South Kore (11) and Japan (37).

Additionally, our subgroup analysis revealed that the association between moderate alcohol consumption and hepatic fibrosis was predominantly present in the overweight/obese or abdominal obesity subgroup, with no significant correlation observed in the lean MASLD subgroup. Previous research has indicated that the combined effects of being overweight or obese and moderate alcohol consumption may hasten the progression of NAFLD. A population-based study in Germany revealed that an alcohol intake of up to 20 g per day was associated with NAFLD in non-obese men (OR = 5.04, 95% CI: 1.16-21.86) (38). By contrast, the same level of alcohol consumption corresponded to an OR of 14.88 (95% CI: 3.55-62.40) in overweight individuals and 35.23 (95% CI: 8.32-149.21) in obese individuals. Another cohort study from Finland found that 1 daily drink of alcohol in men with the highest WHR tertile yielded a similar relative risk for advanced liver disease as 4 daily drinks in other men (39). This suggests that the presence of obesity or central obesity may amplify alcoholic hepatotoxicity. Therefore, for adult males who are overweight, obese, or have abdominal obesity, regardless of the presence of MASLD, we contend that there is no safe level of alcohol consumption.

Our study indicated that non-invasive markers of hepatic fibrosis were significantly higher in MetALD males compared to both non-drinking and moderate-drinking males with pure MASLD. Similarly, a study from primary care settings in Korea reported significantly higher mean magnetic resonance elastography values in the MetALD group than in either the MASLD or alcoholic liver disease (ALD) groups (40). In addition, trend analysis in our study revealed that the risk of hepatic fibrosis in males with MASLD or MetALD increased with higher alcohol intake. A decade-long prospective study involving half a million Chinese individuals demonstrated that regular drinkers exhibited a greater propensity for liver cirrhosis compared to lifelong non-drinkers, following a dose-response pattern (41). Clinical and mechanistic evidence further indicates that alcohol consumption, in conjunction with metabolic abnormalities may accelerate the progression of liver fibrosis (42). Therefore, male patients with MetALD—who experience dual insults from both metabolic dysfunction and increased alcohol intake—are likely to have a greater fibrosis burden than those with pure MASLD.

Our study had several limitations. Firstly, the correlation identified in a cross-sectional study does not necessarily imply causation. Secondly, we diagnosed fatty liver based on ultrasound in this study. While ultrasound is the most commonly used imaging technique for diagnosing fatty liver disease, its moderate sensitivity may lead to underdiagnosis of mild cases (43). Future studies should employ the controlled attenuation parameter (CAP) or ultrasonic attenuation parameter (UAP) based on transient elastography (TE), which offer higher sensitivity for detecting fatty liver (44). Thirdly, we used non-invasive markers to define liver fibrosis in this study. Although less accurate than pathological testing, FIB-4 is widely used as a non-invasive method for screening hepatic fibrosis (11, 25, 27) and has demonstrated good diagnostic accuracy for fibrosis in individuals with MASLD (22) or MetALD (23). For predicting advanced hepatic fibrosis in NASH, FIB-4 ≥ 2.67 has an 80% positive predictive value, and FIB-4 < 1.30 has a 90% negative predictive value (45). In the future, the accuracy of liver fibrosis detection could be enhanced by combining FIB-4 with liver stiffness measurement. Fourthly, this study was conducted in a single center in Southwest China, which limits the generalizability of our findings. Finally, future research should also explore the impact of different types of alcohol consumption, alcohol metabolism genes, and drinking patterns on liver fibrosis in MASLD.

Despite these limitations, our study offers several strengths. Firstly, focusing on a high-risk population—Chinese males, who have the fastest-growing incidence of NAFLD and the highest number of alcohol-attributable deaths globally—our study adopts the latest definition of MASLD and demonstrates that moderate alcohol consumption is detrimental to hepatic fibrosis progression. Our findings provide a scientific basis for alcohol consumption recommendations for Chinese males with MASLD. Secondly, data collection based on health check-ups, combined with a rigorous quality control system, ensured the accuracy and reliability of our data. Thirdly, to minimize confounding effects, we conducted a sub-analysis using propensity score matching followed by logistic regression, enhancing the robustness of our results.

## 5 Conclusion

In China, even moderate alcohol consumption may significantly increase the risk of hepatic fibrosis in males with MASLD who are overweight/obese or have abdominal obesity. Therefore, non-lean males with MASLD may not have a safe level of alcohol consumption and should abstain from alcohol entirely. Moreover, males with MetALD, who consume more alcohol than those with pure MASLD, may face a higher risk of hepatic fibrosis. Thus, strict limitation of alcohol intake is strongly recommended to prevent adverse outcomes.

## Data Availability

The raw data supporting the conclusions of this article will be made available by the authors, without undue reservation.
